# Enhanced analog circuit fault diagnosis via continuous wavelet transform and dual-stream convolutional fusion

**DOI:** 10.1038/s41598-025-02596-6

**Published:** 2025-06-05

**Authors:** Zhiwen Hou, Jingrui Liu, Sijiu Yu

**Affiliations:** 1 Chongqing University-University of Cincinnati Joint Co-op Institute, Chongqing University, Chongqing, 400044 China; 2 Department of Electrical and Computer Engineering, University of Cincinnati, Cincinnati, OH 45221 USA; 3 Hongshen Honors School, Chongqing University, Chongqing, 401331 China

**Keywords:** Analog circuit fault diagnosis, Continuous wavelet transform, Dual-stream convolution, Attention mechanism, Feature extraction, Electronics, photonics and device physics, Information theory and computation, Techniques and instrumentation, Computational science, Computer science, Information technology, Scientific data, Electrical and electronic engineering

## Abstract

Analog circuit fault diagnosis is crucial for ensuring the reliability and safety of electronic systems. To overcome the limitations of traditional methods, this study proposes a novel analog circuit fault diagnosis method based on Continuous Wavelet Transform (CWT) and Dual-Stream Convolutional Neural Network (DSCNN). The method uses CWT to convert raw fault waveform data into two-dimensional time–frequency images and employs a one-dimensional convolutional neural network (1D-CNN) to extract temporal features and a two-dimensional convolutional neural network (2D-CNN) to extract image features, achieving feature fusion. Additionally, the model incorporates a Convolutional Block Attention Module (CBAM), which includes channel and spatial attention modules, to enhance the model’s expressive power. Experiments on the Sallen–Key band-pass filter circuit, four-op-amp biquad high-pass filter circuit, and Tow-Thomas filter circuit validate the effectiveness of the proposed method, demonstrating excellent fault classification accuracy. Their classification accuracies reached 1.0000 ± 0.0000, 99.66% ± 0.0016, and 0.9771 ± 0.0023, respectively. Under various SNR conditions, our proposed model consistently maintains the highest classification accuracy with minimal impact from SNR variations. Furthermore, detailed practical experiments on the four-op-amp biquad high-pass filter circuit show that this model outperforms 1DCNN, CWT-CNN, and ISSA-SVM by 3.85%, 5.50%, and 6.39%, respectively, further proving the model’s superior feature extraction capability.

## Introduction

With the rapid advancement of science and technology, electronic circuits are increasingly utilized in communication, industrial control, medical devices, household appliances, aerospace, military applications, and many other fields^1–3^. Due to the poor tolerance of their components, electronic circuits are more susceptible to interference and external influences. According to relevant surveys, approximately 80% of all electronic circuits used in electronic devices are digital circuits. While digital circuits constitute the majority of electronic circuits, analog circuits still account for only a small portion, roughly 20% of the total, yet they contribute to over 80% of system failures^4^. Compared to digital circuits, analog circuits are more challenging to diagnose and have slower development due to their inherent continuity, nonlinearity, tolerance issues, and high sensitivity to environmental factors^5–7^. Soft faults in analog circuits are primarily caused by abnormal changes in resistance, capacitance, and inductance parameters, making their measurement complex and challenging^8,9^.

The faults in analog circuits are primarily classified into two major categories: catastrophic faults and parametric faults^10^. Catastrophic faults refer to failures that result in the complete cessation of circuit operation. These include open-circuit faults, where components behave as disconnected; short-circuit faults, where component pins or conduction paths come into contact; and grounding faults, where signals or components are shorted to ground. Such faults can lead to circuit failure or abnormal operation and are therefore referred to as hard faults.

In contrast, parametric faults, also known as soft faults, involve abnormal variations in parameters such as resistance, capacitance, and inductance, causing deviations from nominal component values. Unlike hard faults, soft faults exhibit gradual changes, pushing the circuit beyond its normal operating range or producing inaccurate results. Detecting these faults is more complex.

Both catastrophic and parametric faults can damage circuit components and the circuit as a whole. Therefore, identifying and resolving these issues is essential to ensure proper circuit operation. However, due to inherent challenges such as the difficulty in modeling analog circuits, limited test nodes, fault diversity, and component tolerances, effective solutions for diagnosing faults in analog circuits remain insufficient^9,11^.

In the field of analog fault diagnosis, two primary simulation methods are commonly employed: simulation before test (SBT) and simulation after test (SAT)^12^, as depicted in Table 1. These methods play an indispensable role in the design and testing of modern electronic systems.

**Table 1:** System-based testing and system acceptance testing overview.

	SBT	SAT
Definition	Virtual modeling and testing before physical testing to identify and correct design issues, saving time and costs	Virtual modeling after initial physical testing to compare results and optimize performance
Advantages	Early issue detection; Reduced prototyping; Comprehensive design validation	Improved fault diagnosis; Performance optimization; Deeper system understanding
Application scenarios	Early design phase for concept validation and iterative design	Late development phase for prototype testing and product optimization

Benefiting from the rapid advancements in sensor technology and big data storage, machine learning-based methods have become one of the mainstream approaches in analog circuit fault diagnosis^13^. Machine learning algorithms can learn from large datasets and excel in precise fault detection and classification tasks.

Parai et al. proposed a parameter fault diagnosis technique for analog circuits based on multi-source data fusion. This method combines data from multiple sources to enhance accuracy, employing an extended Kalman filter to estimate circuit parameters and diagnose faults^14^.

Laidani et al. applied an adaptive neuro-fuzzy and feature reduction-based fault classification approach for analog circuits. This method extracted various time and frequency domain features from signals for model training^15^. Similarly, Song et al. utilized fractional Fourier transform to extract multiple statistical features and fed the data into an SVM for training^7^.

From this, it can be seen that data-driven methods generally involve two main stages: (1) extracting features of different types of faults in analog circuits, and (2) building models based on the extracted features to complete fault diagnosis. Regarding feature extraction methods, the performance of classifiers depends heavily on the efficiency of the features to ensure high accuracy. Some methods highlight their importance by revealing detailed response signals they can provide. Common feature extraction techniques include Fourier transform^16^, principal component analysis (PCA)^17^, decision tree^18^, and Bayesian network^19^. However, traditional feature extraction methods often struggle to capture transient characteristics or abrupt changes in frequency components, especially in time-domain or frequency-domain analyses. In scenarios requiring high-resolution time–frequency analysis, such as transient feature analysis or unstable signal analysis, these traditional methods are less effective.

In recent years, deep learning has provided a new approach to analog circuit fault diagnosis. Artificial intelligence algorithms are primarily used for automatic feature extraction and fault identification^20,21^. Deep learning algorithms can directly extract deep fault features, significantly improving diagnostic accuracy. Therefore, deep learning algorithms have gained extensive application in fault diagnosis. Yuan et al. proposed a fault diagnosis method based on artificial neural networks (ANN), which selected the entropy and kurtosis of output signals as inputs to the neural network^22^. As an improvement, Zhao et al. adopted deep belief networks (DBN) for fault diagnosis, demonstrating more reliable performance^23^. Similar studies also employed DBN for analog circuit fault analysis^24–27^. However, the fully connected structure of DBN involves numerous network parameters^23^, leading to high modeling costs. While stacked autoencoders can quickly complete fault diagnosis, they struggle to capture the internal representations of the network, making it difficult to observe the feature extraction process.

Shokrolahi et al. proposed a fault detection method based on deep convolutional neural networks, which has strong feature extraction and discrimination capabilities^28^. Additionally, one-dimensional CNNs possess unique abilities for handling one-dimensional time series data and have been widely applied in audio processing^29^, bearing fault diagnosis^30–32^, and structural damage detection^33^. Zhang et al. preprocessed raw time-domain data using a backward interference strategy and employed a novel CNN structure for feature extraction and diagnosis^34^. Yang et al.^13^ proposed a 1D convolutional neural network for analog circuit fault diagnosis, using raw signals as input. Du et al.^35^ developed a CNN-based fault diagnosis method for analog circuits, directly feeding output signals under different fault states into the CNN.

However, 1D-CNNs primarily focus on capturing local features in a single direction, which can lead to the loss of critical information and limit their ability to extract global features that reveal the dependencies in long-sequence signals. Moreover, the feature extraction capabilities of traditional CNNs are limited, often failing to effectively capture the complex nonlinear relationships and dynamic changes in analog circuit signals. This limitation hinders their application in accurately diagnosing faults.

While these studies have addressed certain challenges related to the nonlinearities and tolerances in analog circuit simulations, limitations such as low feature extraction efficiency, insufficient time–frequency resolution, risk of information loss, and inadequate nonlinear feature capture remain. Consequently, developing effective and straightforward feature extraction methods is of significant importance for capturing the fundamental characteristics of analog circuit faults.

To overcome the limitations of traditional methods, this paper proposes a fault diagnosis method for analog circuits based on temporal features and a dual-stream convolutional fusion attention mechanism with CWT. The method converts raw fault waveform data into two-dimensional time–frequency images using CWT, enabling 2D-CNN to extract image features. Simultaneously, the raw data is directly fed into a 1D-CNN to extract temporal features, achieving the fusion of high-dimensional image features and one-dimensional temporal features.

Moreover, the model incorporates a CBAM attention mechanism, which includes channel and spatial attention modules. This mechanism refines the extracted features, enhancing the model’s expressive power. Such a structure not only retains the dynamic properties of temporal information but also fully leverages the spatial information of image features, thereby improving the robustness and generalization ability of the algorithm.

To evaluate the effectiveness of our proposed method, experiments were conducted on two commonly used fault diagnosis circuits: a Sallen–Key band-pass filter circuit and a four-op-amp biquad high-pass filter circuit. The proposed CWT-DSCNN-CBAM method was validated through these experiments. The proposed structure can offer the following novelties and advantages from the computational analysis.

- CWT time–frequency representation provides locally localized features in both time and frequency domains. This enhances training efficiency by making it easier to identify transient characteristics or abrupt changes in frequency components within fault signals, which are challenging to analyze using conventional time-domain or frequency-domain approaches.
- The dual-stream convolution integrates one-dimensional time-series features and two-dimensional wavelet images, leveraging information from different modalities to provide a more comprehensive description of the data’s characteristics. As a result, it enables the extraction of richer and more discriminative features, which improves fault recognition accuracy. The approach only requires raw fault waveform data, which can be transformed into a two-dimensional CWT time–frequency image.
- We introduce the CBAM attention mechanism. CBAM applies dual attention weighting across both the channel and spatial dimensions of the extracted features. This highlights important characteristics and suppresses irrelevant ones, thereby enhancing the model’s discriminative ability.

The organization of this paper is as follows: “Structure design and mathematical model” section introduces the principle of feature extraction based on wavelet transform, analyzes the architecture of the proposed CWT-DSCNN-CBAM model, provides the fundamental equations designed for this method, and analyzes the time complexity of this model. “Simulation analysis and results” section discusses the simulation analysis and practical experiments conducted to evaluate the proposed method, including an introduction to the structure of the test platform and an analysis of the test results, as well as comparisons with mainstream fault detection methods and performance comparisons under different noise levels. Finally, “Conclusion” section summarizes the conclusions drawn from this study.

## Structure design and mathematical model

**Fig. 1:**
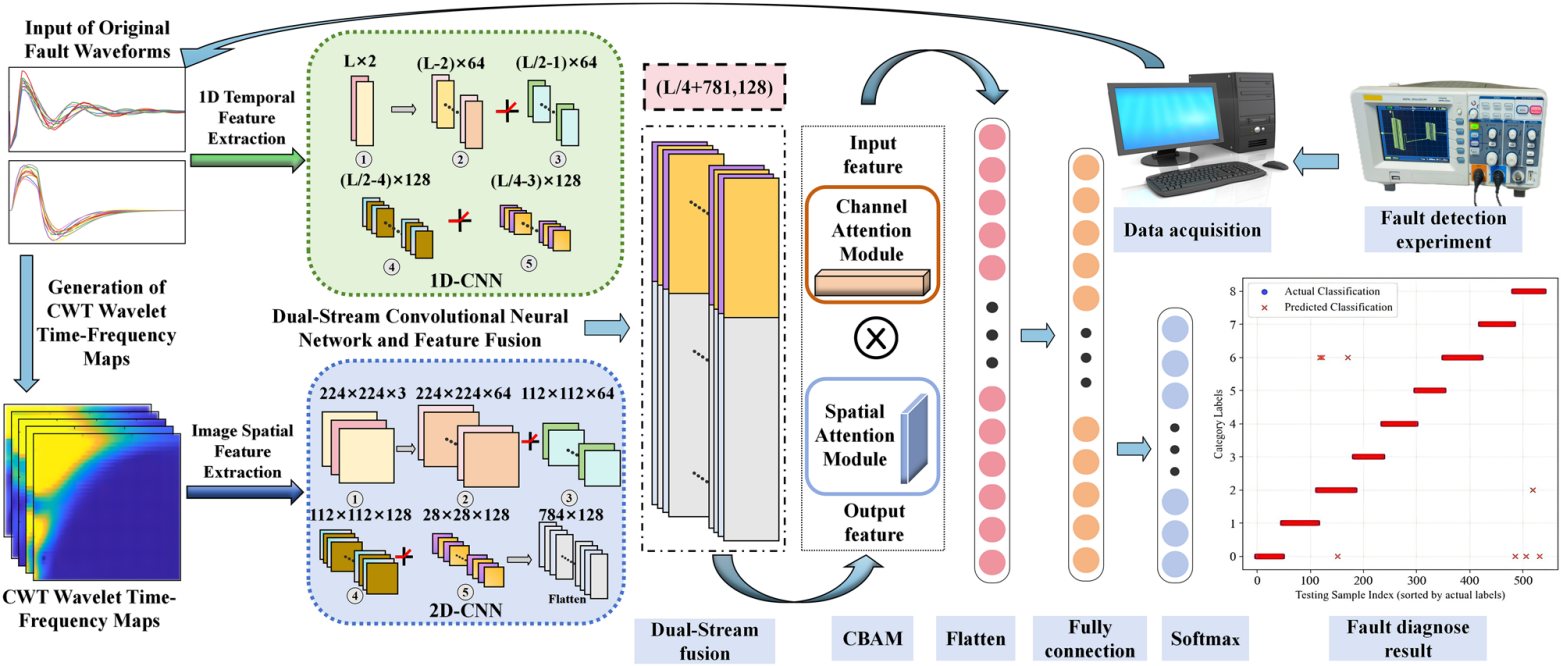
Circuit fault diagnosis model based on dual-stream fusion.

In this section, we delve into the structure design and mathematical model of our proposed CWT-DSCNN-CBAM method, detailing the mathematical formulations and architectural design to illustrate how these components work together to achieve superior fault classification performance. The overall process of the model is shown in Fig. 1

Typically, traditional CNNs have limited feature extraction capabilities and only allow a single type of input data^36^, which can lead to the loss of critical fault-related information. To address this limitation, we redesigned the frameworks of 1D CNN and 2D CNN and incorporated the CBAM to enhance feature representation by introducing attention mechanisms in both the channel and spatial dimensions. The overall framework design is shown in Table 2, which provides a detailed description of 1D-CNN, 2D-CNN, and dual-stream fusion, including the selection and design of detailed parameters such as layer type and neuron count.

**Table 2:** Detailed design of CWT-DSCNN-CBAM.

Detailed design	Layer Type	Filter/Neuron count	Kernel size/Stride	Activation function	Output shape
1DCNN	Input layer				(L, 2)
	Convolutional layer 1	64	3/1	ReLU	(L-2, 64)
	Pooling layer 1		2/2		(L/2 -1, 64)
	Convolutional layer 2	128	3/1	ReLU	(L/2 -4, 128)
	Pooling layer 2		2/2		(L/4 -3, 128)
2DCNN	Input layer				(224,224,3)
	Convolutional layer 1	64	3 × 3/1	ReLU	(224,224,64)
	Pooling layer 1		2 × 2/2		(112,112,64)
	Convolutional layer 2	128	3 × 3/1	ReLU	(112,112,128)
	Pooling layer 2		4 × 4/4		(28,28,128)
	Flatten layer				(784,128)
Dual-stream fusion	Fusion layer				(L/4 + 781, 128)
	Flatten layer				((L/4 + 781) × 128,)
	Fully connected layer 1	120			(120,)
	Fully connected layer 2	84			(84,)
	Output layer	N		Softmax	(N,)

For the 1D CNN framework, the convolution operation for a one-dimensional input signal $x \in R^{L \times C}$ is defined as follows:

1 $$z\left[ {i,j} \right] = \sum\limits_{k = 0}^{K - 1} {w\left[ {j,k} \right] \cdot x\left[ {i + k,j} \right] + b\left[ j \right]}$$

where $L$ represents the length of the time series, $C$ denotes the number of signal channels, and $K$ indicates the size of the convolution kernel. The parameter $w\left[ {i,j} \right]$ corresponds to the $k - th$ convolution kernel in the $j - th$ channel, while $b\left[ j \right]$ is the bias term. The max-pooling operation is employed for dimensionality reduction and the retention of critical local features, where $K_{p}$ represents the size of the pooling window.

2 $$z^{\prime}\left[ {i,j} \right] = \max \left\{ {z\left[ {i + k,j} \right]|k \in \left[ {0,K_{p} } \right)} \right\}$$

After the convolution and max-pooling operations, the feature maps are flattened and fed into a fully connected layer to produce the predicted probabilities for each class. Here, $z_{k}$ represents the activation value for class $k$, and $N$ denotes the total number of classes.

The 1D-CNN is fundamentally similar to the traditional CNN, with the primary difference being that its convolution kernels slide along the time steps^37^, making it well-suited for extracting features from 1D time-series data. Our 1D-CNN structure is specifically designed for time-series fault classification, as detailed in Table 2. It consists of an input layer, two convolutional layers alternated with pooling layers, two fully connected layers, and a final classification layer. The input layer processes data with a shape of (L, 2), representing time and signal values. The first convolutional layer uses kernels of size 3 with 64 channels and applies the ReLU activation function, followed by a pooling layer (pool size 2, stride 2). The second convolutional layer increases the number of channels to 128, with each convolutional layer followed by a pooling layer configured similarly.

For the 2D-CNN framework applied after extracting time–frequency features using CWT, this study utilizes CWT to perform multi-scale decomposition of fault time-series signals, enabling time–frequency analysis of the signals. The definition of the CWT is as follows:

3 $$W_{x} \left( {s,\tau } \right) = \int_{ - \infty }^{ + \infty } {x\left( t \right)} \psi^{*} \left( {\frac{t - \tau }{s}} \right)dt$$

where $x\left( t \right)$ is the input signal, $\psi \left( t \right)$ is the mother wavelet function,$s$ is the scale factor, which controls the “stretching” of the wavelet function, determining its resolution in the frequency domain. Larger scale factors correspond to lower frequencies, allowing the capture of low-frequency components of the signal, while smaller scale factors are associated with higher frequencies. $\tau$ is the time parameter, which controls the translation of the wavelet function on the time axis and determines the time resolution. $\psi^{*} \left( t \right)$ represents the complex conjugate of the mother wavelet function.

In this study, the Morlet wavelet is chosen as the mother wavelet function due to its excellent localization properties in both time and frequency domains. It is non-orthogonal, making it highly suitable for CWT. Additionally, its complex-valued nature allows it to provide both amplitude and phase information of the signal simultaneously, further enhancing its effectiveness in analyzing the transient and frequency components of fault signals. The Morlet wavelet is defined as:

4 $$\psi_{\tau ,s} \left( \tau \right) = \frac{1}{\sqrt s }\pi^{{ - \frac{1}{4}}} e^{{iw_{0} \frac{t - \tau }{s}}} e^{{ - \frac{1}{2}\left( {\frac{t - \tau }{s}} \right)^{2} }}$$

To ensure comprehensive capture of the different frequency components in the fault signal, we employed a multi-scale analysis approach., the range of the scale factor $s$ is set from 1 to 128, increasing with a step size of 2, in order to capture both low-frequency background noise and transient high-frequency components. The time parameter $\tau$ ranges from 0 to the signal length, increasing with the sampling interval as the step size, ensuring comprehensive capture of the transient features of the signal. This enables CWT to generate accurate 2D time–frequency images, providing high-quality input for subsequent feature extraction and laying a solid foundation for fault diagnosis.

The 2D time–frequency images generated by CWT are then input into the 2D-CNN for feature extraction. As shown in Table 2, the 224 × 224 image first passes through the first convolutional layer, which contains 64 filters of size 3 × 3 and a ReLU activation function, to extract low-level local features. This is followed by a 2 × 2 max-pooling operation to reduce the size of the feature map. The feature map then enters the second convolutional layer, which contains 128 filters of size 3 × 3 and another ReLU activation function. This layer further captures higher-level features, and again, a 2 × 2 max-pooling operation is applied to compress the features. Finally, the output from the Flatten layer is a feature map with the same number of channels as the 1D-CNN, enabling further processing.

### CBAM attention mechanism

**Fig. 2:**
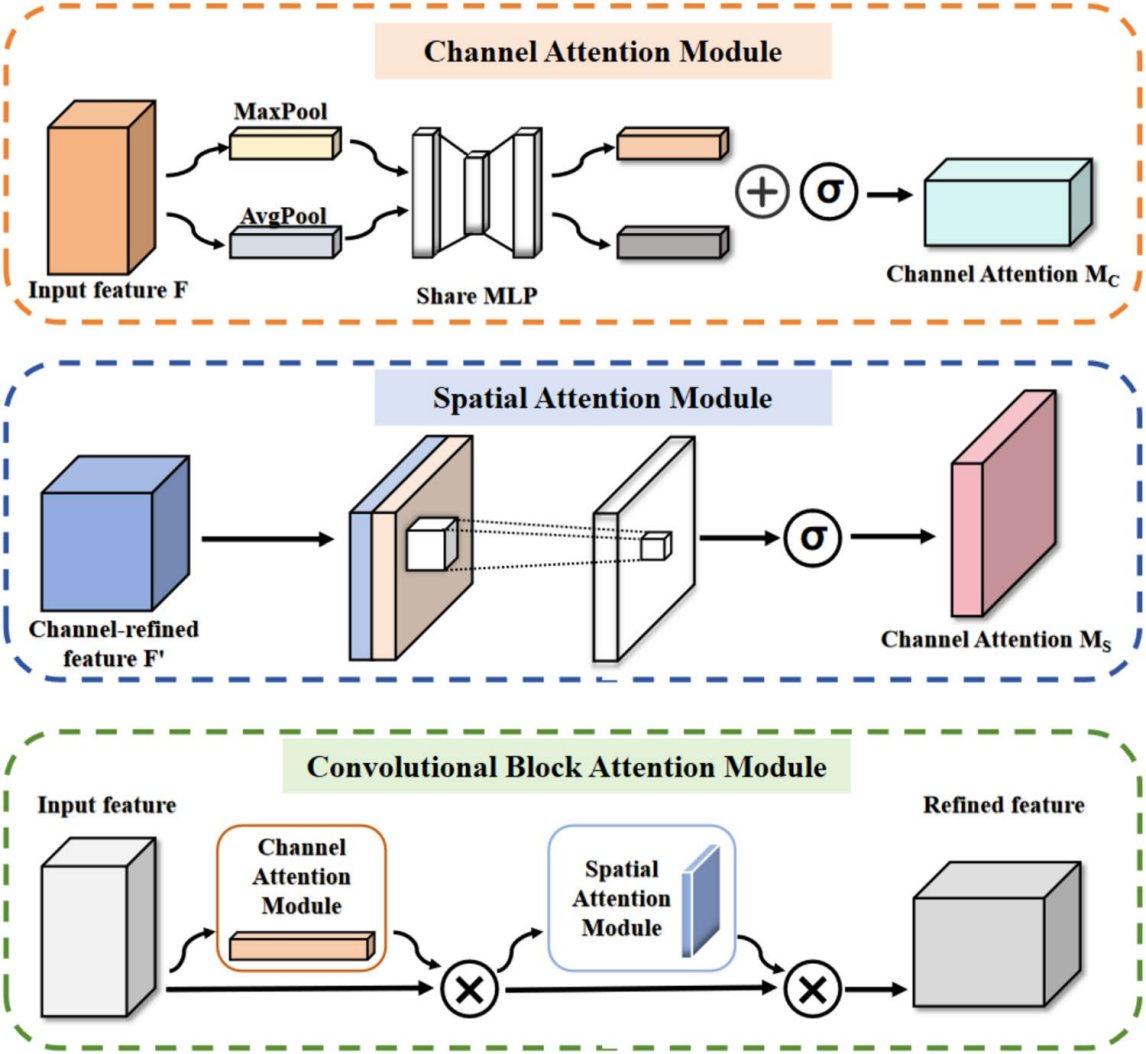
Schematic diagram of CBAM.

To further enhance the model’s expressiveness and optimize the feature extraction process, we introduce the CBAM attention mechanism, as illustrated in Fig. 2 CBAM incorporates both channel and spatial attention mechanisms, enabling more refined weighting of the extracted features. This allows the model to focus effectively on important features while suppressing irrelevant information, thereby improving its ability to distinguish critical fault characteristics. Consequently, the model can selectively highlight the most informative parts of the feature maps, leading to superior fault diagnosis results.

The channel attention mechanism is utilized to reconstruct the input feature map and is subsequently followed by the spatial attention mechanism, which further refines the reconstructed feature map to produce the final feature map. The detailed steps involved in the CBAM attention mechanism are outlined as follows.

#### Step 1 CBAM channel attention operation

The input feature map $F$ is processed through both global average pooling and global max pooling, which generate one-dimensional feature vectors. These vectors are then passed through a shared-weight multi-layer perceptron (MLP) to compute the channel attention map. The MLP consists of two convolution layers: (1) The first convolution layer reduces the feature dimension. (2) The second convolution layer restores the feature dimension back to its original size.

The two resulting feature vectors are added together and then passed through a Sigmoid activation function to generate the channel attention map $M_{{\text{C}}}$, which is used to recalibrate the channel-wise features. The calculation of the channel attention map is given by:

5 $$M_{C} \left( F \right) = \sigma \left( {MLP\left( {AvgPool\left( F \right)} \right) + MLP\left( {MaxPool\left( F \right)} \right)} \right)$$

Simplifying the aforementioned formula, we can derive the following simplified equation:

6 $$M_{C} \left( F \right) = \sigma \left( {W_{1} \left( {W_{0} \left( {F_{avg}^{c} } \right)} \right) + W_{1} W_{0} \left( {F_{\max }^{c} } \right)} \right)$$

#### Step 2 CBAM spatial attention operation

Once the channel-enhanced feature map $F^{\prime}$ is generated, the spatial attention module further emphasizes the prominent regions of the feature map along the spatial dimension. Initially, global average pooling and global max pooling are separately applied to $F^{\prime}$, producing two spatial descriptor maps. These two maps are then concatenated along the channel dimension to form a unified representation. Subsequently, this combined representation undergoes a 7 × 7 convolution operation to capture local spatial dependencies, and is finally processed by a Sigmoid activation function to generate the spatial attention map $M_{{\text{S}}}$.The calculation of the channel attention map is given by:

7 $$M_{S} \left( F \right) = \sigma \left( {{\text{f}}^{7 \times 7} \left[ {AvgPool\left( F \right);MaxPool\left( F \right)} \right]} \right)$$

Simplifying the aforementioned formula, we can derive the following simplified equation:

8 $$M_{S} \left( F \right) = \sigma \left( {f^{7 \times 7} \left[ {F_{avg}^{S} ;F_{\max }^{S} } \right]} \right)$$

#### Step 3 Feature reconstruction

Ultimately, the channel-enhanced feature $F^{\prime}$ is weighted by the spatial attention map $M_{{\text{S}}}$, generating the final enhanced feature $F^{\prime \prime }$. The refined feature map can then be used for subsequent layers of the convolutional neural network, thereby enabling further enhancement and reconstruction of the features.

In the context of complex and diverse fault modes, conventional 1D-CNN and 2D-CNN models encounter limitations in processing multimodal data. To overcome this challenge, this study proposes a circuit fault diagnosis model based on dual-stream fusion. Specifically, the 1D-CNN and 2D-CNN streams generate feature vectors with an equal number of output channels. Within the dual-stream fusion module, the feature vectors from these two streams are concatenated to form a unified feature representation, which are subsequently weighted by the CBAM attention mechanism and then flattened through a flattening layer. Subsequently, the fused features are propagated through two fully connected layers and a Softmax classifier to output the predicted probabilities for fault categories. The model is trained using a cross-entropy loss function, which optimizes its parameters by minimizing the loss, thus facilitating the accurate identification of various fault modes. The corresponding formula is given as follows:

9 $$L = \sum\limits_{C = 1}^{C} {y_{i,c} \log \left( {\hat{y}_{i} ,c} \right)}$$

where $$y_{i,c}$$ is the true label of the sample on the category $$c$$, $$\overset{\lower0.5em\hbox{$\smash{\scriptscriptstyle\frown}$}}{y}_{i,c}$$ is the predicted probability of the category.

### Time complexity analysis

The DSCNN proposed in this study consists of 1D-CNN and 2D-CNN, and classification is performed through feature fusion. In order to evaluate the computational efficiency of the model, we analyze the time complexity of each component, mainly considering the computational amount of convolution operations, pooling operations, and fully connected layers.

#### Computational complexity of 1D-CNN

1D-CNN is mainly used to process time series data, and its computational complexity is mainly determined by the convolution layer. For a 1D sequence with an input length of L, the time complexity of the convolution operation can be expressed as:

10 $$O\left( {Conv1} \right) = O\left( {C_{in} \times C_{out} \times K \times L} \right)$$

where $C_{in}$ and $C_{out}$ are the number of input and output channels, respectively, and $K$ is the size of the convolution kernel. For a two-layer convolution structure, the overall complexity is as follows:

11 $$O\left( {1D - CNN} \right) = O\left( {2 \times C_{in} \times C_{out} \times K \times L} \right)$$

Considering that the computational complexity of pooling operations is relatively low (involving only taking the maximum or average value within a window), it can be considered negligible. Therefore, the computational complexity of 1D-CNN primarily depends on the length of the input sequence $L$ and the number of channels.

#### Computational complexity of 2D-CNN

2D-CNNs are primarily used for extracting image features, and their computational complexity depends on the input size, the number of channels, and the size of the convolution kernels. For an input image of size $H \times W$ with $C_{in}$ channels, the computational complexity of the convolutional layer is as follows:

12 $$O\left( {Conv} \right) = O\left( {C_{in} \times C_{out} \times K_{h} \times K_{w} \times H \times W} \right)$$

In this study, the 2D-CNN employs two convolutional layers, with the computational complexity of the first layer calculated as follows:

13 $$O\left( {Conv1} \right) = O\left( {3 \times 64 \times 3 \times 3 \times 224 \times 224} \right)$$

The computational complexity of the second layer is as follows:

14 $$O\left( {Conv2} \right) = O\left( {64 \times 128 \times 3 \times 3 \times 112 \times 112} \right)$$

Since pooling operations mainly involve dimension reduction with a fixed window, their computational complexity is much lower than that of convolution operations, thus having a minimal impact on the overall computation time and can be neglected. Overall, the computational complexity of the 2D-CNN increases significantly with the increase in input size $H \times W$.

#### Computational complexity of dual-stream fusion

In the DSCNN architecture, the output features from the 1D-CNN and 2D-CNN are concatenated through a fusion layer and then classified by a fully connected layer. The computational complexity of the fully connected layer is determined by the number of neurons, which can be expressed as follows:

15 $$O\left( {FC} \right) = O\left( {N_{in} \times N_{out} } \right)$$

Among them, the computational complexity of the first fully connected layer is as follows:

16 $$O\left( {FC1} \right) = O\left( {\left( {{L \mathord{\left/ {\vphantom {L 4}} \right. \kern-0pt} 4} + 781} \right) \times 128 \times 120} \right)$$

The computational complexity of the second fully connected layer is as follows:

17 $$O\left( {FC2} \right) = O\left( {120 \times 84} \right)$$

The computational complexity of the final output layer is as follows:

18 $$O\left( {Output} \right) = O\left( {84 \times N} \right)$$

From the above analysis, we can see that the computational complexity of 1D-CNN is mainly affected by the input sequence length $L$, while the computational complexity of 2D-CNN increases with the input image size $H \times W$. The computational complexity of the fusion layer is mainly determined by the fully connected layer, and the computational complexity is higher when the input dimension is larger.

In addition, if CBAM is introduced, additional computational overhead will be added. For example, the channel attention part needs to perform global average pooling and full connection calculations, and the additional computational complexity is $O\left(2{C}^{2}\right)$, while the spatial attention part increases the computational complexity $O\left(49C\right)$, which is the number of channels $C$. Under the premise of ensuring the prediction accuracy of the model, DSCNN has better computational efficiency. Although CBAM slightly increases the computational cost, it can effectively enhance the feature extraction capability and improve the classification accuracy. The model achieves a better balance between computational complexity and performance improvement.

## Simulation analysis and results

### Analog circuit fault definition

To rigorously evaluate the efficacy of our novel CWT-DSCNN-CBAM framework, we established comprehensive simulation models incorporating three distinct active filter topologies: (i) Sallen–Key bandpass filter circuit, (ii) four-op-amp biquad high-pass filter circuit, and (iii) Tow-Thomas filter circuit^38^. Specifically, we employed Multisim software, developed by National Instruments (NI), with Application Version 14.0.593, for circuit modeling and the acquisition of raw data. Additionally, we utilized Jupyter Notebook version 5.7.2 and Python environment version 3.12.4 for setting up the deep learning framework, with TensorFlow version 2.18.0 for model training and performance evaluation.

#### *Example 1: *Sallen–*Key bandpass filter circuit*

**Fig. 3:**
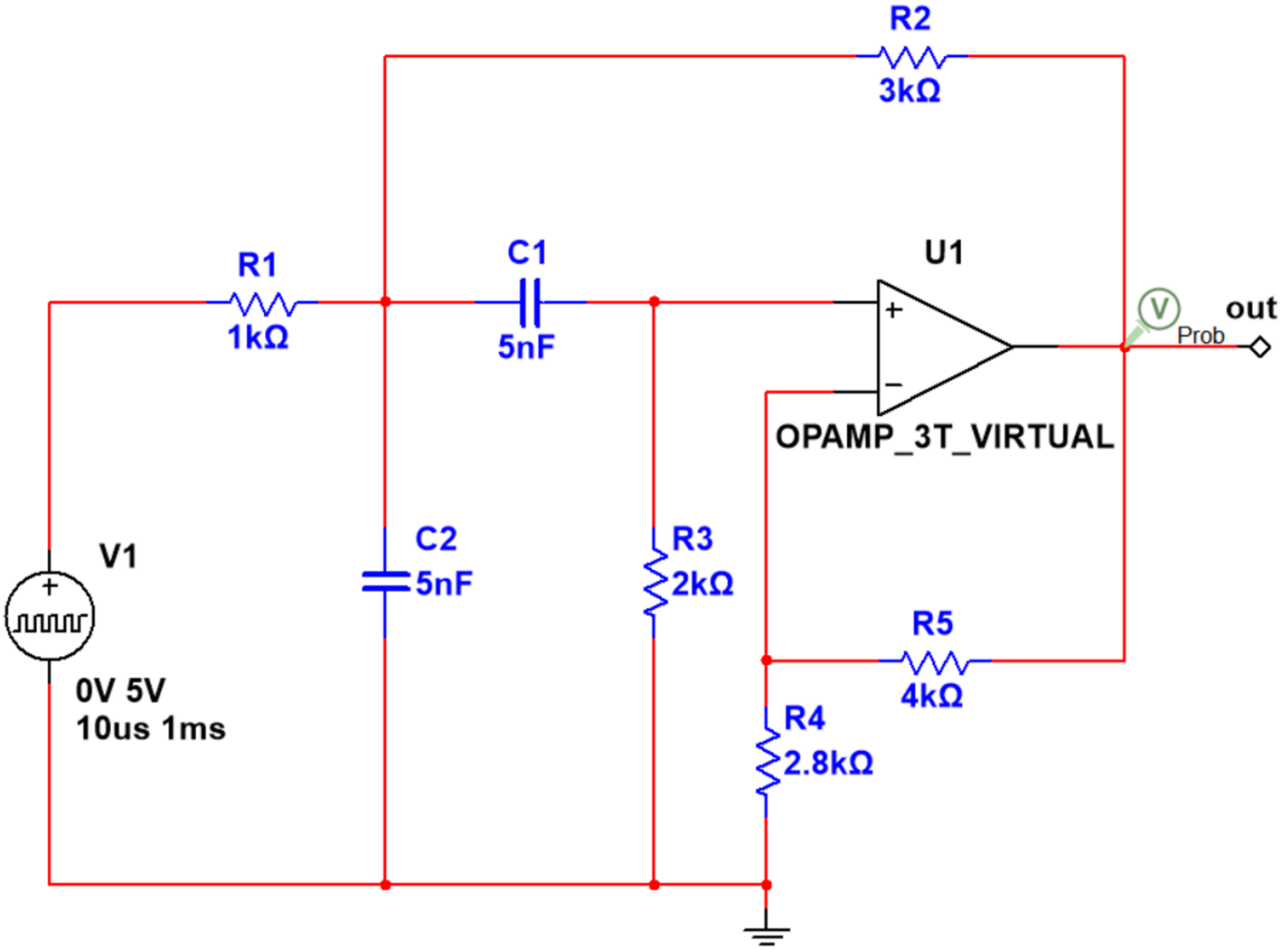
Sallen–Key bandpass filter circuit diagram.

The schematic diagram of the Sallen–Key bandpass filter circuit is shown in Fig. 3. In this circuit, the operational amplifier (U1) serves as the core component, responsible for signal amplification and processing. The circuit includes two 5 nF capacitors, C1 and C2, as well as five resistors: R1 (1 kΩ), R2 (3 kΩ), R3 (2 kΩ), R4 (2.8 kΩ), and R5 (4 kΩ). These components work together to enable the filter to selectively amplify signals within a specific frequency range while suppressing signals outside of this range, both below and above the desired frequencies. The input signal is applied at the V1 port, and the filtered output is obtained at the OUT port.

Taking into account the errors in actual applications, we set a 5% tolerance for resistors and a 10% tolerance for capacitors. The input signal has a pulsed value of 5 V with a duration of 10 μs. This circuit includes both fault-free (NF) and eight distinct fault modes, including C1↓, C1↑, C2↓, C2↑, R2↓, R2↑, R3↓, and R3↑. In our experimental setup, the “↓” denotes a value that is 30% lower than the nominal value, while the “↑” denotes a value that is 30% higher than the nominal value, with specific details provided in Table 3.

**Table 3:** Detailed description of Sallen–Key bandpass filter fault modes.

Fault type	Fault mode	Nominal value	Tolerance range	Fault value
F00	NF	–	–	–
F01	C1↓	5 nF	10%	3.5 nF
F02	C1↑	5 nF	10%	6.5 nF
F03	C2↓	5 nF	10%	3.5 nF
F04	C2↑	5 nF	10%	6.5 nF
F05	R2↓	3kΩ	5%	2.1 kΩ
F06	R2↑	3kΩ	5%	3.9 kΩ
F07	R3↓	2kΩ	5%	1.4 kΩ
F08	R3↑	2kΩ	5%	2.6 kΩ

**Fig. 4:**
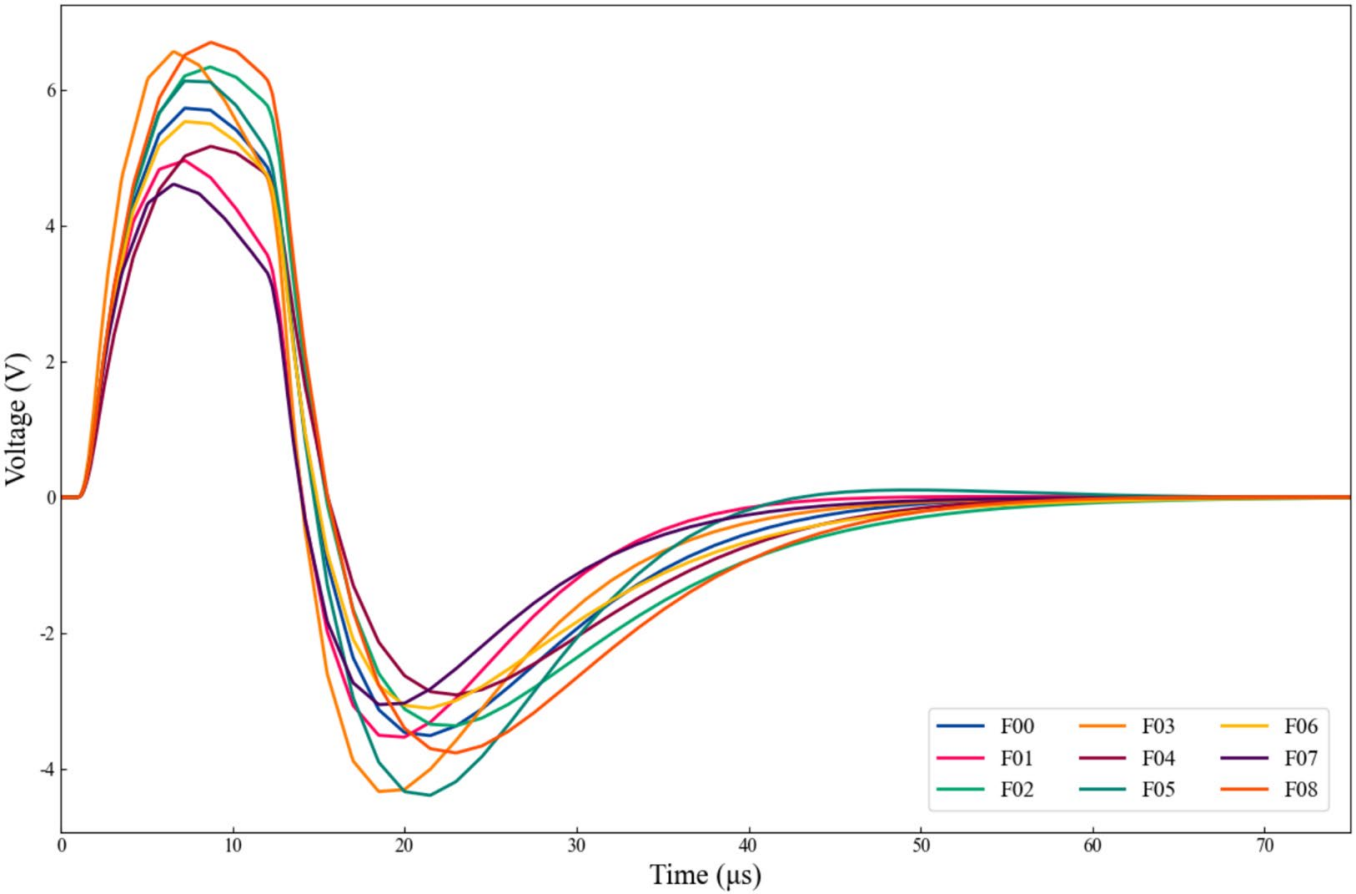
Output response of Sallen–Key bandpass filter under different fault mode**s**.

Figure 4 illustrates the system’s time-domain response signal after the application of the aforementioned pulsed signal. As shown in the figure, the oscillation of the voltage waveform primarily occurs within the first 75 μs. Therefore, we selected the sample set within this range and uniformly sampled 750 data points, with a sampling interval of 0.1 μs. Additionally, we conducted 200 Monte Carlo simulations by introducing variations in the resistor and capacitor values within their tolerance range. The same procedure was adopted in the following examples and will not be reiterated.

#### Example 2: Four-op-amp biquad high-pass filter circuit

**Fig. 5:**
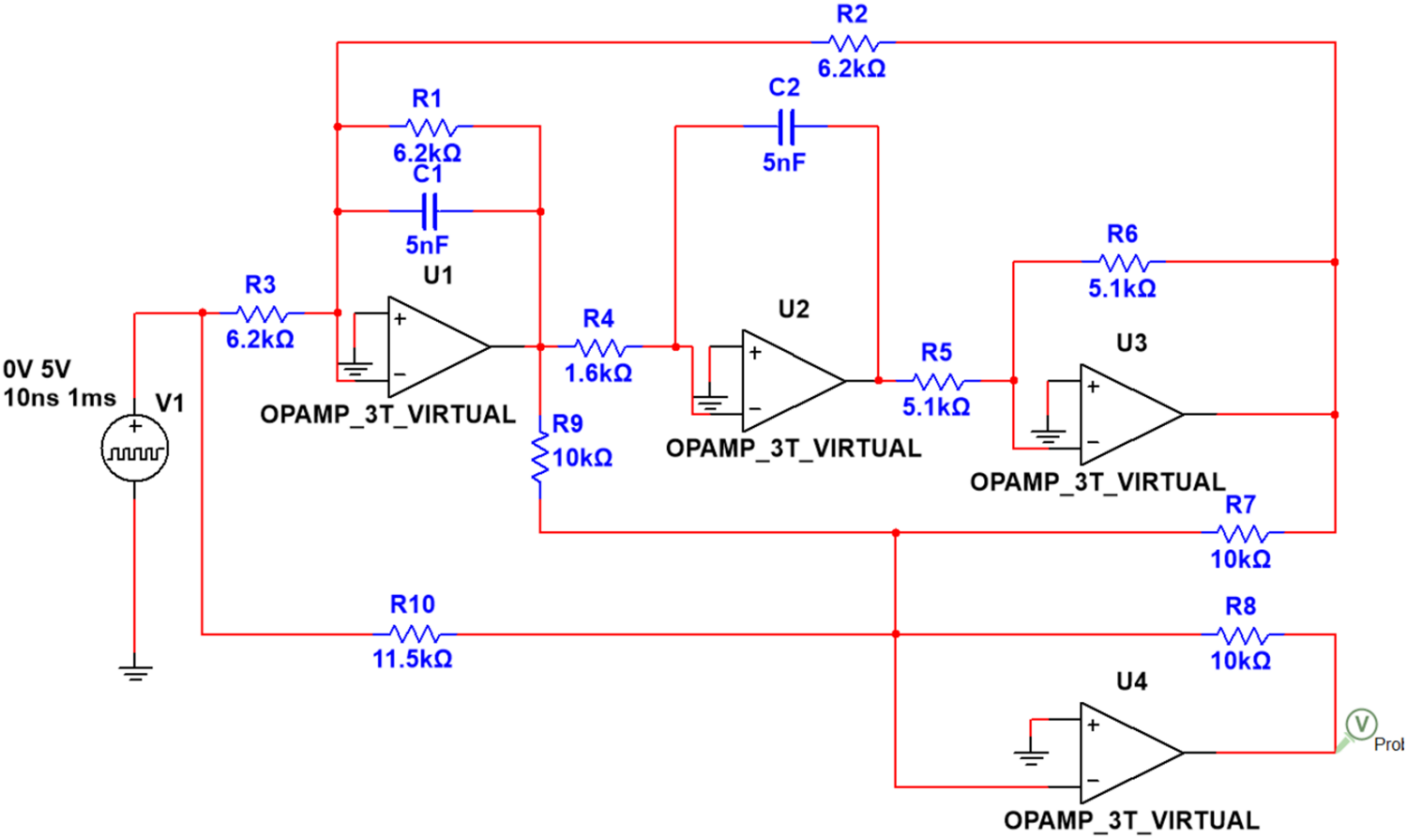
Four-op-amp biquad high-pass filter circuit diagram.

To further validate the accuracy and superiority of CWT-DSCNN-CBAM in more complex circuits, we utilized the biquad high-pass filter circuit, which is composed of four operational amplifiers, as shown in Fig. 5. This circuit consists of four operational amplifiers, namely U1, U2, U3, and U4. The resistors and capacitors in the circuit (e.g., R1, R2 = 6.2 kΩ; R5, R6 = 5.1 kΩ; R4 = 1.6 kΩ; R7, R8, R9 = 10 kΩ; R10 = 11.5 kΩ; C1, C2 = 5 nF) determine the filtering characteristics, with the output signal being provided by U4. The circuit is designed to amplify high-frequency signals while suppressing low-frequency signals.

As in Example 1, the tolerance for resistors and capacitors is set to 5% and 10%, respectively. The input pulsed value is 5 V, with a duration of 10 μs. In addition to the normal non-fault (NF) case, this circuit includes 12 distinct fault categories, including C1↓, C1↑, C2↓, C2↑, R1↓, R1↑, R2↓, R2↑, R3↓, R3↑, R4↓, and R4↑, which are specifically described in Table 4.

**Table 4:** Detailed description of four-op-amp biquad high-pass filter fault modes.

Fault type	Fault mode	Nominal value	Tolerance range	Fault value
F00	NF	–	–	–
F01	C1↓	5 nF	10%	3.5 nF
F02	C1↑	5 nF	10%	6.5 nF
F03	C2↓	5 nF	10%	3.5 nF
F04	C2↑	5 nF	10%	6.5 nF
F05	R1↓	6.2 kΩ	5%	4.3 kΩ
F06	R1↑	6.2 kΩ	5%	8.1 kΩ
F07	R2↓	6.2 kΩ	5%	4.3 kΩ
F08	R2↑	6.2 kΩ	5%	8.1 kΩ
F09	R3↓	6.2 kΩ	5%	4.3 kΩ
F10	R3↑	6.2 kΩ	5%	8.1 kΩ
F11	R4↓	1.6 kΩ	5%	1.1 kΩ
F12	R4↑	1.6 kΩ	5%	2.1 kΩ

**Fig. 6:**
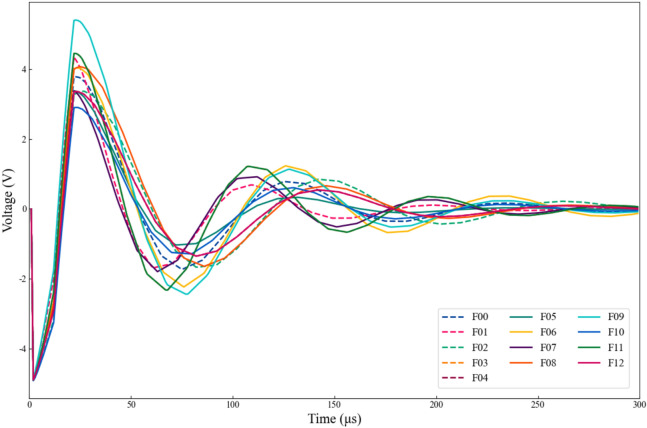
Output response of four-op-amp biquad high-pass filter under different fault modes.

Figure 6 shows the system’s time-domain response signal after applying the above-mentioned pulsed signal. In contrast to Example 1, we selected data from the 0-300μs range as the sample set, with 300 uniformly sampled data points at a sampling interval of 1 μs.

#### Example 3: Tow-Thomas filter circuit

**Fig. 7:**
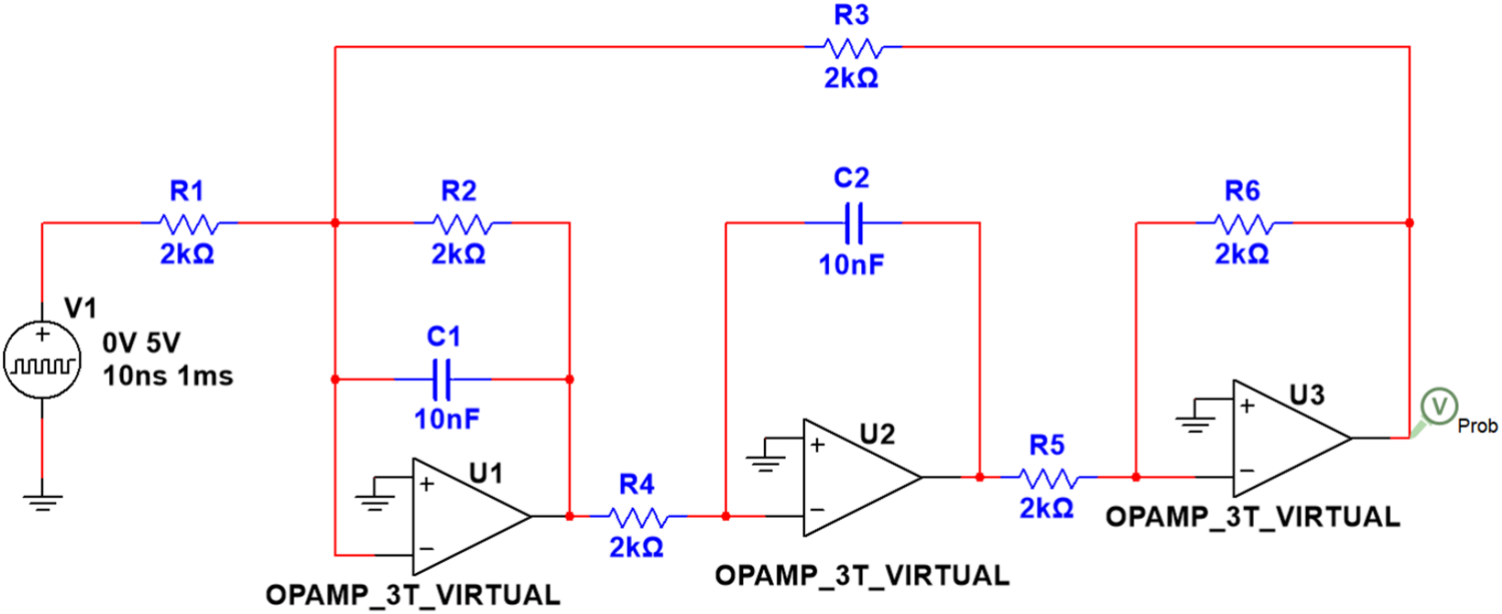
Tow-Thomas filter circuit diagram.

As illustrated in Fig. 7, the circuit diagram represents a Tow-Thomas filter, which is a double second-order filter composed of three operational amplifiers. This example will demonstrate the diagnostic capabilities of the algorithm under both single and multiple fault conditions within the load circuit. The filter is constructed with operational amplifiers U1, U2, and U3. The characteristics of the filter are determined by resistors R1 through R6, all of which are set to 2kΩ, and capacitors C1 and C2, which are set to 5nF. The output signal is provided by amplifier U3.

The simulation of the Monte Carlo analysis and the tolerance settings of the resistors and capacitors in the circuit remain the same as before. We have formulated a single fault set comprising 11 states, including NF, C1↓, C1↑, C2↓, C2↑, R3↓, R3↑, R4↓, R4↑, R5↓, R5↑. For detailed parameter settings, please refer to Table 5.

**Table 5:** Detailed description of Tow-Thomas filter circuit single fault modes.

Fault type	Fault mode	Nominal value	Tolerance range	Fault value
F00	NF	–	–	–
F01	C1↓	5 nF	10%	3.5 nF
F02	C1↑	5 nF	10%	6.5 nF
F03	C2↓	5 nF	10%	3.5 nF
F04	C2↑	5 nF	10%	6.5 nF
F05	R3↓	2 kΩ	5%	1.65 kΩ
F06	R3↑	2 kΩ	5%	2.35 kΩ
F07	R4↓	2 kΩ	5%	1.65 kΩ
F08	R4↑	2 kΩ	5%	2.35 kΩ
F09	R5↓	2 kΩ	5%	1.65 kΩ
F10	R5↑	2 kΩ	5%	2.35 kΩ

Furthermore, we have defined a composite fault set^39^, which includes 7 states: C1↑C2↑, C1↓C2↓, C2↑R3↑, C2↓R3↑, R3↑R5↑, R3↑R5↑C1↑, R3↑C1↑C2↓. Detailed information regarding these configurations is presented in Table 6.

**Table 6:** Detailed description of Tow-Thomas filter circuit composite fault modes.

Fault type	Fault mode	Nominal value	Tolerance range	Fault value
F11	C1↑C2↑	5 nF, 5 nF	10%, 10%	6.5 nF, 6.5 nF
F12	C1↓C2↓	5 nF, 5 nF	10%, 10%	3.5 nF, 3.5 nF
F13	C2↑R3↑	5 nF, 2 kΩ	10%, 5%	6.5 nF, 2.35 kΩ
F14	C2↓R3↑	5 nF, 2 kΩ	10%, 5%	3.5 nF, 2.35 kΩ
F15	R3↑R5↑	2 kΩ, 2 kΩ	5%, 5%	2.35 kΩ, 2.35 kΩ
F16	R3↑R5↑C1↑	2 kΩ, 2 kΩ, 5 nF	5%, 5%, 10%	2.35 kΩ, 2.35 kΩ, 6.5 nF
F17	R3↑C1↑C2↓	2 kΩ, 5 nF, 5 nF	5%, 10%, 10%	2.35 kΩ, 6.5 nF, 3.5 nF

**Fig. 8:**
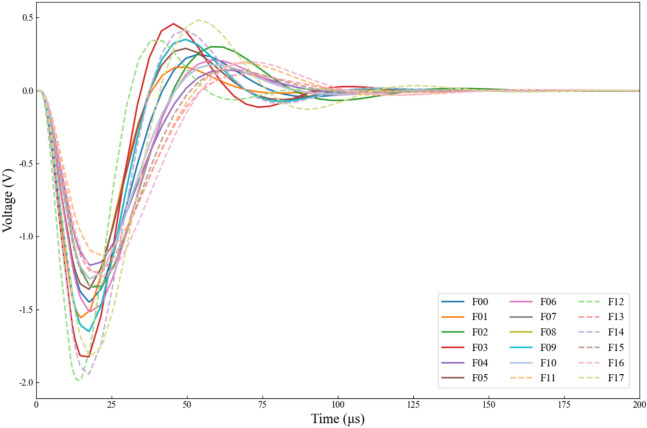
Output response of Tow-Thomas filter under different fault modes.

Figure 8 shows the system’s time-domain response signal after applying the above-mentioned pulsed signal, where the solid line is the signal of normal state and single fault condition, and the dashed line is the signal of multiple fault condition. In contrast to Example 1 and 2, we selected data from the 0–200 μs range as the sample set, with 400 uniformly sampled data points at a sampling interval of 0.5μs.

#### Standardized processing

To optimize neural network training, we normalized the collected data from each sample using min–max standardization, and the ratio of the training set to the test set was set at 7:3. Its mathematical expression is as follows:

19 $$X = \frac{{x_{i} - x_{\min } }}{{x_{\max } - x_{\min } }}$$

### Feature extraction and visualization analysis

**Fig. 9:**
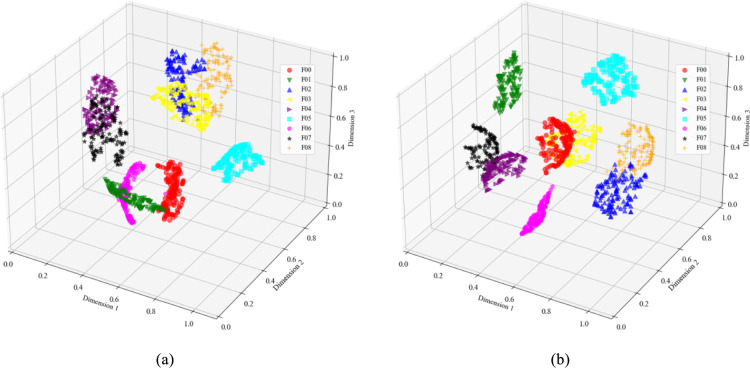
Visualization of Sallen–key bandpass filter circuit feature extraction results. (**a**) Original data of circuit fault; (**b**) FC layer output of CWT-DSCNN-CBAM.

The T-SNE method was used to perform dimensionality reduction and visualize the fault data of the Sallen–Key bandpass filter circuit in three dimensions. Figure 9a shows that the clustering of the original data is not clearly distinguishable, especially with overlap observed between F01 and F06, F02 and F03, and F04 and F07. Figure 9b demonstrates that the features extracted using wavelet transform dual-stream convolution make the data points of different fault modes more distinctively clustered. The three-dimensional scatter points for most fault modes are completely separated, indicating that CWT-DSCNN-CBAM effectively extracts features that differentiate faults.

**Fig. 10:**
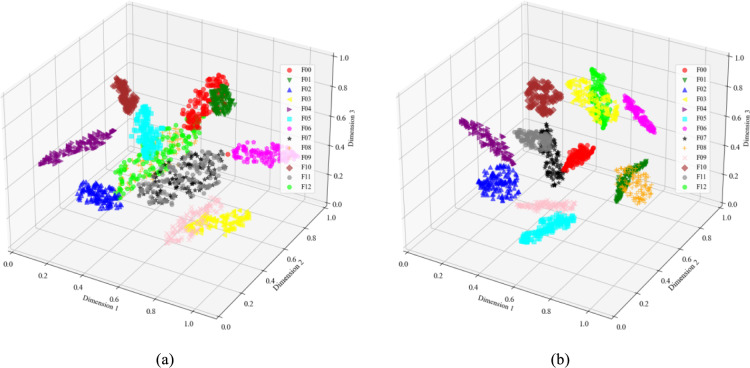
Visualization of four-op-amp biquad high-pass filter circuit feature extraction results. (**a**) Original data of circuit fault; (**b**) FC layer output of CWT-DSCNN-CBAM.

Figure 10 presents the visualization results of the Four-op-amp biquad high-pass filter circuit. In the original data, seven out of the twelve fault types exhibit noticeable overlapping of feature clusters. In contrast, the output classification results from the fully connected (FC) layer of CWT-DSCNN-CBAM model show that the intra-cluster distances are small and the distribution is tightly grouped. Moreover, the distances between different fault types are large, making them easier to distinguish and thus providing strong support for fault diagnosis.

**Fig. 11:**
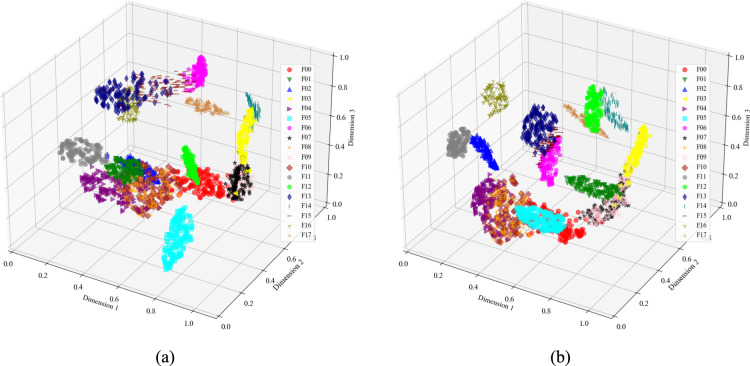
Visualization of Tow-Thomas filter circuit feature extraction results. (**a**) Original data of circuit fault; (**b**) FC layer output of CWT-DSCNN-CBAM.

Figure 11 shows the results of the Tow-Thomas filter circuit feature extraction. In the original data, many of the 18 fault types show obvious feature cluster overlap. Compared with Fig. 11a, the distribution of features processed by the CWT-DSCNN-CBAM model in three-dimensional space is clearer and more separated. Data points of different fault types are more closely clustered together, forming a more obvious clustering effect, especially for specific clusters of fault diagnosis such as F04, F11, F12, and F13, which are more closely clustered. In addition, the distances between different fault types are significantly increased, effectively improving the accuracy and reliability of fault diagnosis.

### Experimental results and evaluation metrics

**Fig. 12:**
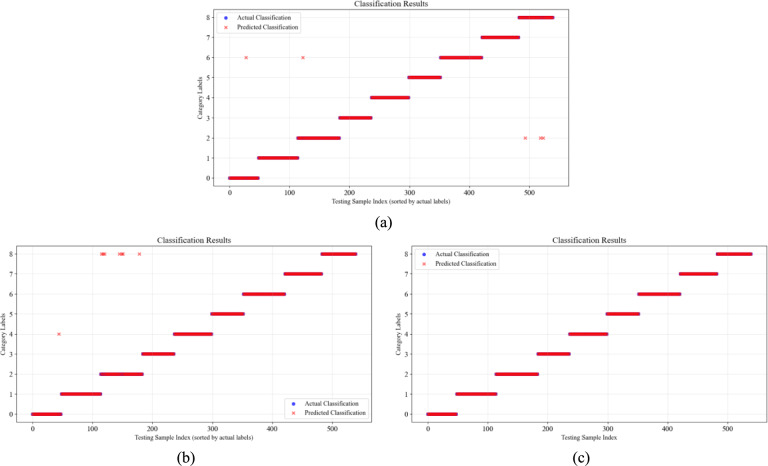
Fault classification results of Sallen–Key bandpass filter circuit. (**a**) 1DCNN; (**b**) CWT-CNN; (**c**) Ours.

To further validate the classification accuracy of CWT-DSCNN-CBAM model, we visualized the actual labels and the classification labels predicted by the model. Figure 12a and b show the classification results using the 1DCNN and CWT-CNN models respectively. Figure 12c presents the classification results of the wavelet transform dual-stream convolution model, demonstrating that the model successfully identifies all fault categories in the Sallen–Key bandpass filter circuit.

**Fig. 13:**
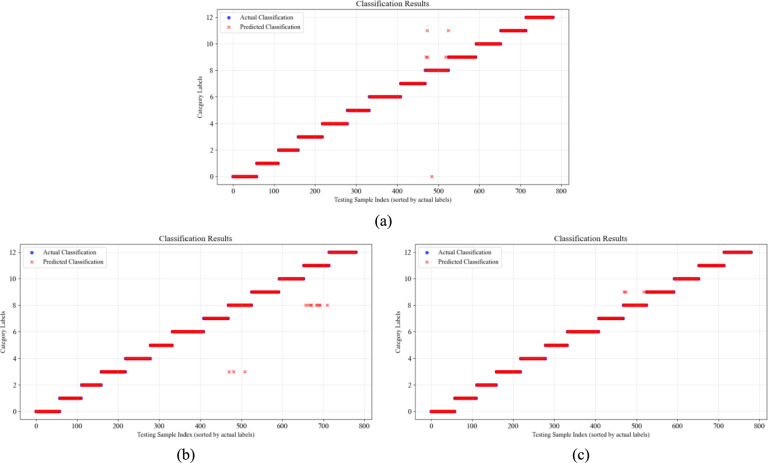
Fault classification results of four-op-amp biquad high-pass filter circuit. (**a**) 1DCNN; (**b**) CWT-CNN; (**c**) Ours.

For the more complex four-op-amp biquad high-pass filter circuit shown in Fig. 13, only F08 contains a few misclassified data points, while the accuracy of the 1D-CNN and CWT-CNN models shows a significant reduction.

**Fig. 14:**
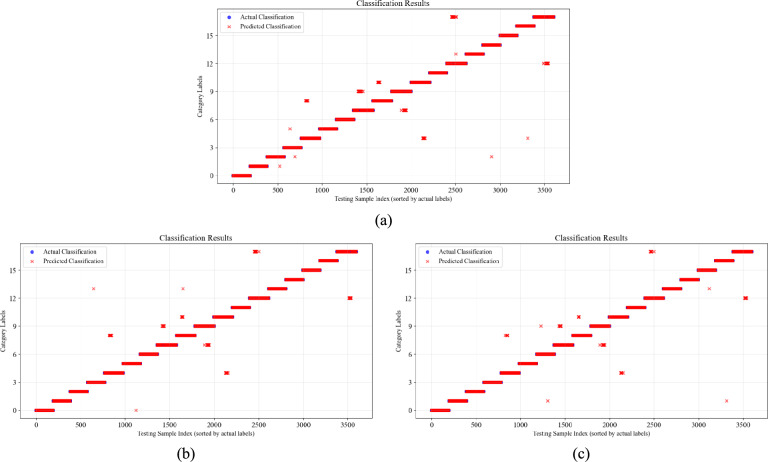
Fault classification results of Tow-Thomas filter circuit. (**a**) 1DCNN; (**b**) CWT-CNN; (**c**) Ours.

Figure 14 shows the fault classification detection results of the Tow-Thomas component circuit. When faced with complex fault conditions where single faults and multiple faults coexist, the 1DCNN model and the CWT-CNN model have a relatively low degree of match between the actual classification and the predicted classification, and there are many prediction errors. In contrast, the CWT-DSCNN-CBAM model we proposed maintains a correct classification rate of more than 97.5%, and the number of misclassified samples between the main error-prone categories such as F07 and F09, and F12 and F17 is significantly lower than that of other methods. There are almost no errors in the classification of F00 to F06.

Table 7 compares the accuracy of the proposed model with mainstream fault detection methods. In all experiments, for a fair comparison, the 1D-CNN was configured with two convolutional layers (64 and 128 filters with a kernel size of 3) and followed by a max-pooling layer for each. This was followed by a flatten layer, a fully connected layer with 128 units, a dropout layer with a rate of 0.2, and an output layer corresponding to the number of classes, with a softmax activation function for multi-class classification. CWT-CNN model used 64 3 × 3 convolution kernels in the first layer with 2 × 2 pooling, and 128 3 × 3 convolution kernels with ReLU activation in the second layer, followed by 2 × 2 pooling. This was followed by a Flatten layer and a fully connected layer with 128 neurons. The LSTM network consisted of two hidden layers, each with 128 hidden units^40^. SVM selects RBF as the kernel function^1^. In addition, to demonstrate the superiority of our model, we also compared two mainstream state-of-the-art methods in the field, namely the ISSA-SVM proposed in reference^41^ and ELM based method proposed in reference^42^. We used classification accuracy as the performance metric, conducted 10 repeated experiments, and reported the mean and standard deviation. The results show that our method achieves an improvement of 0.4% to 7.6% compared to the baseline, with the smallest standard deviation, demonstrating superior diagnostic capabilities for faults in complex circuits.

**Table 7:** Model performance evaluation for different circuit faults (mean ± std).

Circuit	1DCNN	CWT-CNN	LSTM	SVM	ISSA-SVM	ELM based method	Ours
Sallen–Key bandpass filter	0.9919 ± 0.0028	0.9835 ± 0.0022	0.9764 ± 0.0078	0.9752 ± 0.0064	0.9828 ± 0.0030	0.9796 ± 0.0055	1.0000 ± 0.0000
Four-op-amp biquad high-pass filter	0.9920 ± 0.0025	0.9834 ± 0.0048	0.9342 ± 0.0092	0.9259 ± 0.0113	0.9437 ± 0.0025	0.9381 ± 0.0038	0.9966 ± 0.0016
Tow-Thomas filter circuit	0.9504 ± 0.0058	0.9623 ± 0.0076	0.9102 ± 0.0040	0.9210 ± 0.0085	0.9629 ± 0.0055	0.9557 ± 0.0082	0.9771 ± 0.0023

**Fig. 15:**
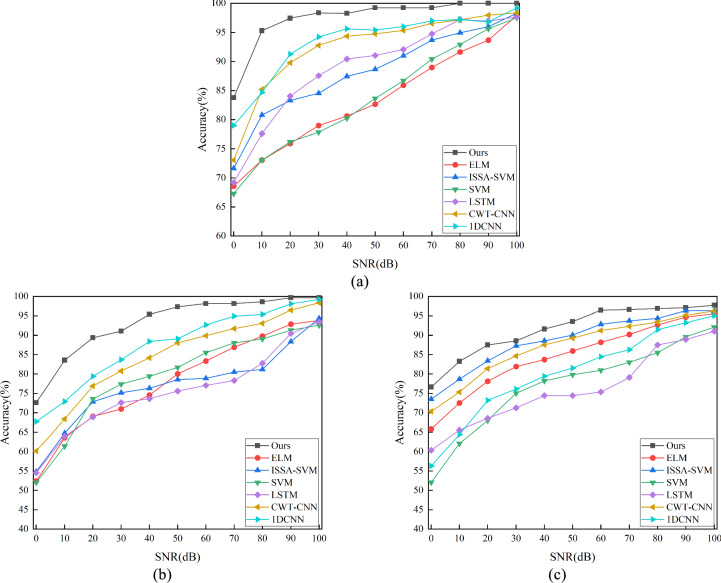
Classification accuracy curve under different SNR. (**a**) Sallen–Key bandpass filter circuit; (**b**) Four-op-amp biquad high-pass filter circuit; (**c**) Tow-Thomas filter circuit.

In actual application environments, internal circuits are often affected by various noises, so the noise resistance of the fault diagnosis model is crucial^43,44^. In this paper, we first define the signal-to-noise ratio (SNR), and its calculation formula is as follows.

20 $$SNR = 10 \lg \frac{{P_{s} }}{{P_{n} }}$$

where ${P}_{s}$ represents the signal power and ${P}_{n}$ denotes the noise power. To evaluate the performance of the proposed model under different noise levels, we conducted experiments and plotted the classification accuracy variation curve over a SNR range of 0 to 100 dB, as shown in Fig. 15. It can be observed from the figure that the classification accuracy exhibits a distinct upward trend with the increase of SNR. Under various SNR conditions, our proposed model consistently maintains the highest classification accuracy with the least impact from SNR variations. For instance, in Fig. 15c, in the Tow-Thomas filter circuit with mixed faults, our model achieves a classification accuracy of 97.71% under low noise interference (SNR = 100 dB), and 76.63% under high noise interference (SNR = 0 dB), demonstrating the highest precision and robustness. In contrast, the second-best ISSA-SVM shows a variation range from 96.29 to 73.51%; whereas the SVM performs the worst, with an accuracy of only 52.07% at SNR = 0 dB. This is attributed to the fact that wavelet transform can separate the useful information of the signal from noise by analyzing the signal at different scales, combined with the feature extraction capability of dual-stream convolution for waveform, thereby endowing it with strong anti-interference capability against circuit noise. Similar results are also reflected in the Sallen–Key bandpass filter and the four-op-amp biquad high-pass filter.

**Fig. 16:**
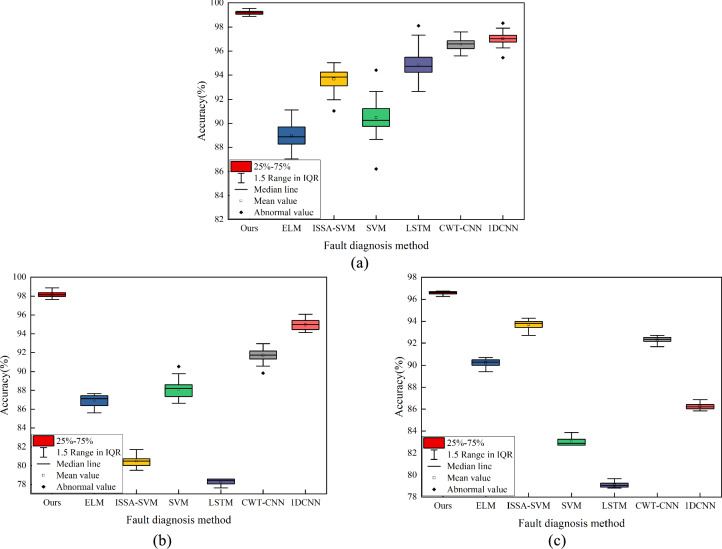
Box plot of fault diagnosis results. (**a**) Sallen–Key bandpass filter circuit; (**b**) Four-op-amp biquad high-pass filter circuit; (**c**) Tow-Thomas filter circuit.

To further explore the model’s stability in real noise environments, we conducted 50 repeated trials under a SNR of 70 dB for three different methods and compared their performance. Specifically, we recorded the highest accuracy achieved every 10 trials. Figure 16a, b, and c show the stability levels of the Sallen–Key bandpass filter circuit, the four-op-amp biquad high-pass filter circuit, and the Tow-Thomas filter circuit using the proposed CWT-DSCNN-CBAM model and six other models including 1DCNN and LSTM, etc. It is evident from the figures that the proposed model has the narrowest interval between the lower and upper quartiles and the most concentrated range within 1.5 IQR in multiple trials. Its distribution is concentrated, with no outliers in the three major categories of experiments. This indicates that the method exhibits the best stability, with the smallest fluctuation in accuracy and the largest mean. This shows that the model can maintain high diagnostic accuracy when facing noise interference.

The experimental results of adjusting the SNR further verified that the proposed model has excellent noise resistance performance under different noise levels. Especially under low SNR conditions, the model can still maintain a high diagnostic accuracy, which provides strong support for its application in actual complex environments.

### Practical experiment

**Fig. 17:**
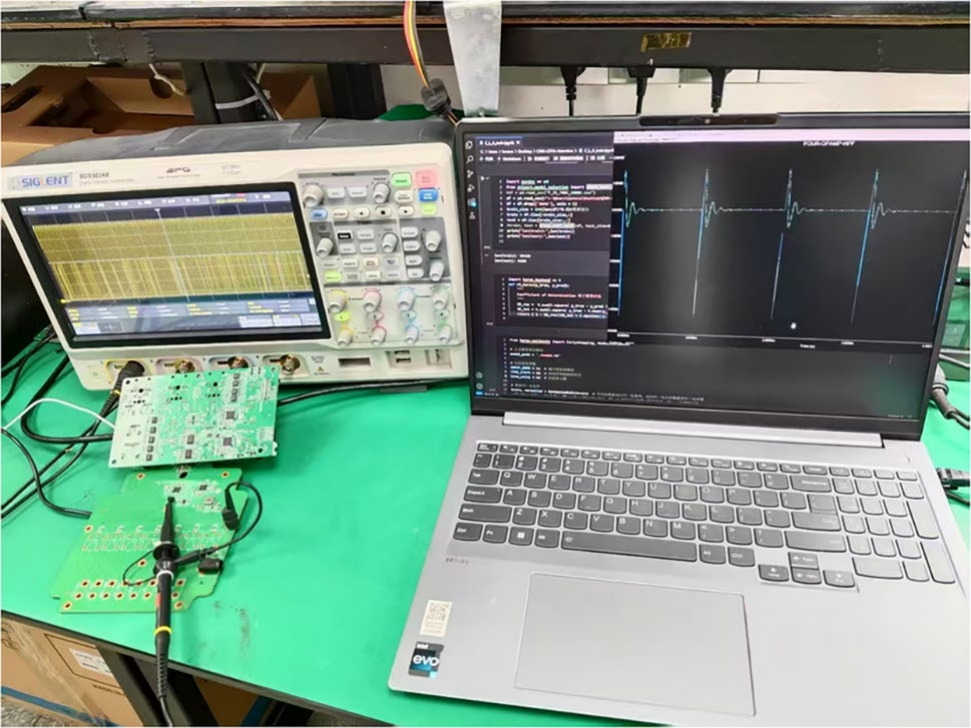
The fault diagnosis experimental platform.

To verify the effectiveness and practicality of the proposed fault diagnosis method for analog circuits based on CWT and dual-stream convolution fusion, this paper conducts detailed physical experiments on a four-op-amp biquad high-pass filter circuit. The experimental platform consists of several key components: a SIGLENT SDG5032X arbitrary waveform generator, a digital storage oscilloscope from SIGLENT, model SDS5034X, an FPGA controller, a power supply, the circuit under test (CUT), and its PCB board. The arbitrary waveform generator is used to produce a standard pulse excitation signal (5 V, 10 $\upmu$s pulse width) as the input signal. The digital oscilloscope is used to monitor and display the output waveform of the circuit in real time for observation and analysis. The FPGA controller is responsible for coordinating the entire testing process, including signal generation and data acquisition, ensuring the automation and accuracy of the testing process. In the experiment, the CUT is a physical circuit designed and built according to the four-op-amp biquad high-pass filter circuit, with adjustable resistors and capacitor interfaces reserved to simulate different fault states, as shown in the aforementioned Fig. 5 and Table 4, and the physical diagram is shown in Fig. 17.

During the data collection phase, 200 samples were collected for each fault mode, with each sample containing 300 data points. The collected signals were normalized using the Max-Min method to facilitate subsequent feature extraction and model training. The ratio of the training set to the test set was set at 7:3. As shown in Table 8, the proposed model achieved the highest classification accuracy, verifying the effectiveness of its improved structure and the feasibility of physical experiments. In comparison, the classification accuracy of SVM, ELM, and LSTM was lower. The improvements of this model over 1DCNN, CWT-CNN, and ISSA-SVM in physical experiments were 3.85%, 5.50%, and 6.39%, respectively. Further analysis found that the classification accuracy of fault types F08-F12 was relatively low across all models, indicating a higher misclassification rate for these categories. This may be due to the greater similarity between these fault patterns, making it difficult to effectively distinguish features, or because these patterns themselves have more complex spatiotemporal characteristics. However, CWT-DSCNN-CBAM still performed better than other models in these categories, indicating its superior feature extraction capability.

**Table 8:** Classification accuracy for different faults types in practical experiment.

Fault type	1DCNN	CWT-CNN	LSTM	SVM	ISSA-SVM	ELM based method	Ours
F00	0.995	0.99	0.985	0.98	0.98	0.995	1
F01	0.99	0.99	0.985	0.985	0.985	0.985	1
F02	0.995	0.99	0.99	0.985	0.975	0.99	1
F03	1	0.99	1	0.98	0.985	0.985	1
F04	0.99	1	0.99	0.995	0.995	0.985	1
F05	0.995	0.985	0.995	0.98	0.99	0.99	1
F06	0.985	0.98	0.975	0.98	0.985	0.985	0.995
F07	0.98	0.995	0.985	0.97	0.985	0.995	1
F08	0.915	0.865	0.74	0.765	0.81	0.695	0.965
F09	0.885	0.855	0.82	0.745	0.77	0.74	0.975
F10	0.925	0.85	0.725	0.735	0.89	0.77	0.98
F11	0.86	0.825	0.815	0.685	0.855	0.785	0.99
F12	0.87	0.855	0.81	0.725	0.85	0.765	0.98
Overall	0.9527	0.9362	0.9088	0.8854	0.9273	0.8973	0.9912

## Conclusion

In this study, we proposed an analog circuit fault diagnosis method based on CWT and DSCNN with attention mechanism. This method was validated on the Sallen–Key band-pass filter, the four-op-amp biquad high-pass filter, and the Tow-Thomas filter. It achieved accuracies of 100%, 99.66%, and 97.71% respectively. This shows its strength and dependability. Compared to traditional fault detection techniques such as 1D-CNN, CWT-CNN, LSTM, SVM, as well as state-of-the-art methods like ISSA-SVM and ELM-based approaches, the CWT-DSCNN-CBAM method showed superior performance, with improvements in accuracy ranging from 0.4 to 7.6%. Experiments under various SNR conditions consistently demonstrated that our proposed model maintained the highest classification accuracy, with minimal impact from SNR variations. Under an SNR of 70 dB, we conducted 50 trials on three different methods and compared their performance, recording the highest accuracy every ten trials. The results indicated that our model exhibited the narrowest interquartile range and the most concentrated distribution across multiple trials, with no outliers, showing optimal stability and minimal fluctuation in accuracy. Furthermore, physical experiments showed that our model outperformed 1DCNN, CWT-CNN, and ISSA-SVM by 3.85%, 5.50%, and 6.39%, respectively, further proving its superior feature extraction capabilities. These results highlight the method’s advantages, including high precision, robustness, and generalization ability, making it a promising tool for analog circuit fault diagnosis across various practical applications, including communications, industrial control, medical devices, and aerospace. The DSCNN, while ensuring model prediction accuracy, offers better computational efficiency. Although CBAM slightly increases computational cost, it effectively enhances feature extraction capabilities and improves classification accuracy. The model achieves an optimal balance between computational complexity and performance improvement.

## Data Availability

All data, models, and code generated or used during the study are available from the corresponding author upon reasonable request.

## References

[1] Su, X. et al. Application of DBN and GWO-SVM in analog circuit fault diagnosis. *Sci. Rep.* **11**, 7969 (2021). 33846418 10.1038/s41598-021-86916-6 PMC8041839

[2] Ji, L., Fu, C. & Sun, W. Soft fault diagnosis of analog circuits based on a ResNet with circuit spectrum Map. *IEEE Trans. Circuits Syst.* **I**(68), 2841–2849 (2021).

[3] Yang, C. Multiple soft fault diagnosis of analog filter circuit based on genetic algorithm. *IEEE Access* **8**, 8193–8201 (2020).

[4] Binu, D. & Kariyappa, B. S. A survey on fault diagnosis of analog circuits: Taxonomy and state of the art. *AEU-Int. J. Electron. C.* **73**, 68–83 (2017).

[5] Aminian, M. & Aminian, F. Neural-network based analog-circuit fault diagnosis using wavelet transform as preprocessor. *IEEE Trans. Circuits Syst.* **II**(47), 151–156 (2000).

[6] Tadeusiewicz, M., Hałgas, S. & Kuczyński, A. New aspects of fault diagnosis of nonlinear analog circuits. *Int. J. Electron. Telecommun.* **61**, 83–93 (2015).

[7] Song, P., He, Y. & Cui, W. Statistical property feature extraction based on FRFT for fault diagnosis of analog circuits. *Analog. Integr. Circuits Signal Process.* **87**, 427–436 (2016).

[8] Xu-Sheng, G., Hong, Q., Xiang-Wei, M., Chun-Lan, W. & Jie, Z. Research on ELM soft fault diagnosis of analog circuit based on KSLPP feature extraction. *IEEE Access* **7**, 92517–92527 (2019).

[9] Arabi, A., Bourouba, N., Belaout, A. & Ayad, M. An accurate classifier based on adaptive neuro-fuzzy and features selection techniques for fault classification in analog circuits. *Integration* **64**, 50–59 (2019).

[10] Yuan, X. et al. Fault diagnosis of analog circuits based on IH-PSO optimized support vector machine. *IEEE Access* **7**, 137945–137958 (2019).

[11] Xie, X. et al. Analog circuits soft fault diagnosis using Rényi’s entropy. *J. Electron Test* **31**, 217–224 (2015).

[12] Arabi, A. et al. An efficient method for faults diagnosis in analog circuits based on machine learning classifiers. *Alex. Eng. J.* **77**, 109–125 (2023).

[13] Yang, H., Meng, C. & Wang, C. Data-driven feature extraction for analog circuit fault diagnosis using 1-D convolutional neural network. *IEEE Access* **8**, 18305–18315 (2020).

[14] Parai, M., Srimani, S., Ghosh, K. & Rahaman, H. Multi-source data fusion technique for parametric fault diagnosis in analog circuits. *Integration* **84**, 92–101 (2022).

[15] Laidani, I. & Bourouba, N. Analog circuit fault classification and data reduction using PCA-ANFIS technique aided by K-means clustering approach. *Adv. Electr. Comp. Eng.* **22**, 73–82 (2022).

[16] Griffiths, P. R. & De Haseth, J. A. *Fourier Transform Infrared Spectrometry* (Wiley, 2007). 10.1002/047010631X.

[17] Jang, K. & Yang, K. Improving principal component analysis (PCA) in automotive body assembly using artificial neural networks. *J. Manuf. Syst.* **20**, 188–197 (2001).

[18] Cui, Y., Shi, J. & Wang, Z. Analog circuit fault diagnosis based on quantum clustering based multi-valued quantum fuzzification decision Tree (QC-MQFDT). *Measurement* **93**, 421–434 (2016).

[19] Aminian, M. & Aminian, F. A modular fault-diagnostic system for analog electronic circuits using neural networks with wavelet transform as a preprocessor. *IEEE Trans. Instrum. Meas.* **56**, 1546–1554 (2007).

[20] Chen, Y., Wu, W. & Li, J. Adaptive attention-enhanced Yolo for wall crack detection. *Appl. Sci.* **14**, 7478 (2024).

[21] Wu, W. et al. Adaptive patch contrast for weakly supervised semantic segmentation. *Eng. Appl. Artif. Intell.* **139**, 109626 (2025).

[22] Yuan, L., He, Y., Huang, J. & Sun, Y. A new neural-network-based fault diagnosis approach for analog circuits by using kurtosis and entropy as a preprocessor. *IEEE Trans. Instrum. Meas.* **59**, 586–595 (2010).

[23] Zhao, G. et al. A novel approach for analog circuit fault diagnosis based on deep belief network. *Measurement* **121**, 170–178 (2018).

[24] Wang, H., Wei, J. & Li, P. Research on fault diagnosis technology based on deep learning. *J. Phys. Conf. Ser.* **2187**, 012041 (2022).

[25] Moezi, A. & Kargar, S. M. Simultaneous fault localization and detection of analog circuits using deep learning approach. *Comput. Electr. Eng.* **92**, 107162 (2021).

[26] Zhong, T., Qu, J., Fang, X., Li, H. & Wang, Z. The intermittent fault diagnosis of analog circuits based on EEMD-DBN. *Neurocomputing* **436**, 74–91 (2021).

[27] Yang, Y., Wang, L., Chen, H. & Wang, C. An end-to-end denoising autoencoder-based deep neural network approach for fault diagnosis of analog circuit. *Analog. Integr. Circuits Signal Process.* **107**, 605–616 (2021).

[28] Shokrolahi, S. M. & Karimiziarani, M. A deep network solution for intelligent fault detection in analog circuit. *Analog Integr. Circ. Sig. Process* **107**, 597–604 (2021).

[29] Hoshen, Y., Weiss, R. J. & Wilson, K. W. Speech acoustic modeling from raw multichannel waveforms. In *2015 IEEE International Conference on Acoustics, Speech and Signal Processing (ICASSP)* 4624–4628 10.1109/ICASSP.2015.7178847. (IEEE, South Brisbane, Queensland, Australia, 2015).

[30] Li, C. et al. A novel bearing fault diagnosis of raw signals based on 1D residual convolution neural network. In *2020 International Conference on High Performance Big Data and Intelligent Systems (HPBD&IS)* 1–6 10.1109/HPBDIS49115.2020.9130567. (IEEE, Shenzhen, China, 2020).

[31] Eren, L., Ince, T. & Kiranyaz, S. A generic intelligent bearing fault diagnosis system using compact adaptive 1D CNN classifier. *J. Signal Process Syst.* **91**, 179–189 (2019).

[32] Peng, D., Liu, Z., Wang, H., Qin, Y. & Jia, L. A novel deeper one-dimensional CNN with residual learning for fault diagnosis of wheelset bearings in high-speed trains. *IEEE Access* **7**, 10278–10293 (2019).

[33] Abdeljaber, O. et al. 1-D CNNs for structural damage detection: Verification on a structural health monitoring benchmark data. *Neurocomputing* **275**, 1308–1317 (2018).

[34] Zhang, C., Zha, D., Wang, L. & Mu, N. A novel analog circuit soft fault diagnosis method based on convolutional neural network and backward difference. *Symmetry* **13**, 1096 (2021).

[35] Du, T., Zhang, H. & Wang, L. Analogue circuit fault diagnosis based on convolution neural network. *Electron. lett.* **55**, 1277–1279 (2019).

[36] Hou, X. et al. Bolt-loosening detection using 1D and 2D input data based on two-stream convolutional neural networks. *Materials* **15**, 6757 (2022). 36234106 10.3390/ma15196757 PMC9572207

[37] Wang, Y. et al. Tight sandstone reservoir classification based on 1DCNN-BLSTM with conventional logging data. *Acta Geophys.*10.1007/s11600-024-01506-0 (2024).

[38] Zhou, X., Tang, X. & Liang, W. A novel analog circuit fault diagnosis method based on multi-channel 1D-resnet and wavelet packet transform. *Analog. Integr. Circuits Signal Process.* **121**, 25–38 (2024).

[39] Jia, Z. et al. A fault diagnosis strategy for analog circuits with limited samples based on the combination of the transformer and generative models. *Sensors* **23**, 9125 (2023). 38005513 10.3390/s23229125 PMC10674503

[40] Han, L., Liu, F. & Chen, K. Analog circuit fault diagnosis using a novel variant of a convolutional neural network. *Algorithms* **15**, 17 (2021).

[41] Wang, G., Tu, Y. & Nie, J. An analog circuit fault diagnosis method using improved sparrow search algorithm and support vector machine. *Rev. Sci. Instrum.* **95**, 055110 (2024). 38743574 10.1063/5.0210515

[42] Biswas, S., Mahanti, G. K. & Chattaraj, N. Investigation of extreme learning machine-based fault diagnosis to identify faulty components in analog circuits. *Circuits Syst. Signal Process.* **43**, 711–728 (2024).

[43] Fang, X., Qu, J., Chai, Y. & Liu, B. Adaptive multiscale and dual subnet convolutional auto-encoder for intermittent fault detection of analog circuits in noise environment. *ISA Trans.* **136**, 428–441 (2023). 36371262 10.1016/j.isatra.2022.10.031

[44] He, W., Yuan, Z., Yin, B., Wu, W. & Min, Z. Robust locally linear embedding and its application in analogue circuit fault diagnosis. *Meas. Sci. Technol.* **34**, 105005 (2023).

